# Obtaining Nanoparticles of Chilean Natural Zeolite and its Ion Exchange with Copper Salt (Cu^2+^) for Antibacterial Applications

**DOI:** 10.3390/ma12132202

**Published:** 2019-07-09

**Authors:** Judith Vergara-Figueroa, Serguei Alejandro-Martín, Héctor Pesenti, Fabiola Cerda, Arturo Fernández-Pérez, William Gacitúa

**Affiliations:** 1 Centro de Biomateriales y Nanotecnología, Universidad del Bío-Bío, Concepción 4030000, Chile; 2 Departamento de Ingeniería en Maderas, Facultad de Ingeniería, Universidad del Bío-Bío, Concepción 4030000, Chile; 3 Nanomaterials and Catalysts for Sustainable Processes Group (NanoCat*p*PS), Concepción 4030000, Chile; 4 Departamento de Procesos Industriales, Universidad Católica de Temuco, Temuco 4780000, Chile; 5 Departamento de Ingeniería en Alimentos, Universidad del Bío-Bío, Chillán 3780000, Chile; 6 Departamento de Física, Facultad de Ciencias, Universidad del Bío-Bío, Concepción 4030000, Chile

**Keywords:** chilean natural nanozeolite, copper salts, ion exchange, antibacterial properties

## Abstract

This article describes the production of nanoparticles of Chilean natural zeolite, using three size reduction methods: Ball mill, microgrinding, and microfluidization. Morphological characterization of samples indicated an average diameter of 37.2 ± 15.8 nm of the zeolite particles. The size reduction and chemical treatments did not affect the morphology or integrity of the zeolite. An increase of the zeolite samples’ Si/Al ratio was observed after the acid treatment and was confirmed by SEM-EDX analysis. Moreover, the effectiveness of the copper salt ion exchange (Cu^2+^) to the zeolite nanoparticles was analyzed by SEM-EDX. XRD analysis indicated that clinoptilolite and mordenite are the main phases of Chilean natural zeolite, and the crystalline structure was not affected by the modification processes. The FTIR characterization showed the presence of chemical bonds of copper with the zeolite nanoparticle framework. The ion-exchanged zeolite nanoparticles were evaluated for antibacterial behavior by the disc diffusion method. Additionally, the minimum inhibitory concentration and minimum bactericidal concentration were obtained. Microbiological assays with copper-exchanged nanozeolites showed an antimicrobial activity with a bactericidal effect against *Escherichia coli* and *Staphylococcus aureus*, which are the primary pathogens of food and are also resistant to multiple drugs. In this study, a new application for natural nanozeolites is demonstrated, as the incorporated copper ions (Cu^2+^) in nanozeolites registered a productive antibacterial activity.

## 1. Introduction

Nanoscale materials are attracting more and more attention due to their superior properties, such as increased surface area and unique structural properties [1]. These properties are displayed to scale, and their applications are endless.

There is great interest in the development of safe and environmentally friendly antimicrobial nanomaterials. Natural inorganic materials, such as zeolites, are good candidates for the design of antimicrobial agents due to their thermal stability, long-lasting action, and chemical resistance [2].

Chile has significant reserves of zeolites of volcanic origin in its territory [3]. Among their main properties are a high degree of hydration, low density, cation exchange capability, electrical conductivity, adsorption of gases and steam, and catalytic properties [4].

One of the advantages of zeolite nanoparticles is their larger external surface area, which is of great importance in several processes, including adsorption and catalysis. Moreover, the proportion of available atoms on the surface increases as the size of the crystal decreases; a particle of 20 nm has around 10% of its available atoms on the surface. This characteristic shows that it is necessary to have a nanoparticle size to obtain a large surface area, leading to the use of zeolite nanoparticles for the preparation of films and membranes as one of its main applications. The small size of nanozeolites offers a high homogeneity and integrity in the films and reduces the number of defects, such as cracks and pores [5]. The application of zeolite in nanometric size makes it possible to increase their surface area, leaving the Brønsted and Lewis active acid sites exposed, generating greater interaction of the molecules with active sites and decreasing the diffusion path lengths between the interstices of the zeolite framework [5,6]. Inside the natural zeolite framework, compensation cations, such as sodium, can be replaced by other metal cations. The rationale for using zeolite as a support for metal ions is that this mode allows continuous resistance against microbial growth, due to the controlled migration of metal ions on the medium in which the zeolite particles are found. This action is feasible due to the ion exchange capacity of the zeolite [7].

In the 1990s, the use of natural zeolite as a cation support (e.g., antibacterial agents in food contact films) was investigated. The ion exchange is carried out by replacing the sodium by other cations, such as copper. The nanozeolites particles activated with copper salt ions (Cu^2+^) can be incorporated directly into a food contact film. The purpose is to allow the retention of ions in the zeolite framework, thus functioning as a carrier. The ions must be retained inside the structure of the zeolite in order to perform the antimicrobial activity. Natural zeolite by themselves are not effective antibacterials, but function as the support of ions that can be used as an antibacterial agent [8].

While several investigations report the exchange of silver or copper ions with zeolite to be used as antibacterial material [2,9,10], it is necessary to mention the disadvantages of the use of silver ions. Ag^+^ is an expensive metal and Ag^+^ is not stable in aqueous solution and tends to be reduced to Ag^0^ after being exposed to light or heat. Ag^+^ also reacts with Cl^−^, HS^−^, SO_4_^2−^, H_2_S, and other anions commonly existing in natural water and moist conditions, forming insoluble compounds, which can result in the loss of its antibacterial activity [11,12]. Copper-charged zeolites are an alternative to the use of zeolites exchanged with silver, since they also have significant effects against microorganisms [12].

Milenkovic et al. (2017) compared the antibacterial activity of natural zeolite and synthetic zeolite A, doped with Cu (II), Zn (II), and Ag (I) ions, against *Escherichia coli*. The results indicated that the type of metal, and not the zeolite type, has a role in antibacterial activity. Zeolites doped with copper ions showed antibacterial activity against *Escherichia coli* [2].

Alswat et al. (2017) investigated the use of synthetic zeolite A with copper oxide applied as antimicrobial material. Their results indicated that zeolite A-copper oxide nanocomposites showed antibacterial activity against Gram-negative and Gram-positive bacteria [13].

At low concentrations, copper is an essential metal for living organisms, but it is toxic to most microorganisms, preventing bacterial growth [14]. Nanomaterials based on copper particles are of particular interest because they have been shown to have negligible sensitivity to human tissues but high sensitivity to microorganisms [15]. Copper ions (Cu^2+^) can act as a bacteriostatic when oscillating in concentrations between 25 to 150 μM, and as a bactericide in concentrations higher than 150 μM. This indicates that the activity toward microorganisms is a function of their concentration. Both metallic copper nanoparticles and copper oxide nanoparticles are active against different pathogens that cause health infections. The incorporation of copper nanoparticles on inert organic matrices has been investigated to produce multifunctional composite materials with antimicrobial activity to be applied to the superficial wounds of patients. The antimicrobial activity is due to the inhibition of protein synthesis, alteration of the cellular membrane of the microorganism, and alteration or destruction of the nucleic acids of bacteria and viruses [16].

Other explanations regarding the biocidal action of metal ions indicate that the difference in charge between bacterial membranes and nanoparticles with metal oxides leads to electrostatic attraction. They accumulate on the surface of the bacteria, altering the structure and permeability of the cell membrane. Gram-negative bacteria have a higher negative charge than Gram-positive bacteria. Therefore, electrostatic interaction will be stronger in Gram-negative strains. The pores of bacterial membranes are of the order of nanometers. Consequently, smaller particle sizes of the biocidal and higher surface areas lead to a better efficiency of the nanostructured metal oxides. The matrices of nanoparticles with metal oxides can slowly release metal ions through adsorption, dissolution, and hydrolysis. These ions are toxic and abrasive to bacteria and, therefore, lyse the cells [17,18].

In Chile, copper is one of the available primary resources, reaching almost 40% of the planet reserves. At the industrial level, the synthesis of copper salt (copper nitrate) is carried out using copper mineral disintegration. It is currently one of the most widely used minerals in the industry, including as a bactericidal material [4]. Copper ions and copper complexes have many applications, such as liquid sterilizer, antibacterial, antifungal, antiviral, and antifouling [19].

The novelty of this research is the obtainment of Chilean natural nanozeolite and its uses as a carrier of antibacterial agents. This nanomaterial, doped with copper ions (Cu^2+^), has chemical stability, low cost, and is environmentally friendly. Its presentation in a nanometric size helps to increase its surface area, allowing applicability in different matrices. This research presents the processes of size reduction and chemical treatments of zeolite. Its morphology, EDX analysis, crystallinity, and union of the zeolite with copper salt ions (Cu^2+^) were evaluated. Also, possible uses as a carrier of antibacterial agents were determined.

## 2. Materials and Methods

### 2.1. Materials

Chilean natural zeolite was obtained from Minera Formas, Chile. Chemical solutions were prepared with ultrapure water. The reagents used were HCl, NaOH, and Cu(NO_3_)_2_·3H_2_O, obtained from Merck. For the microbiological assays, American Type Culture Collection (ATCC) strains were used from the collection unit of the School of Food Engineering of the Universidad del Bío-Bío, Chillán. The strains used were *Escherichia coli* (*E. coli*) and *Staphylococcus aureus* (*S. aureus*). Trypticase broth (30% glycerol) and Müller Hilton agar were used as media to determine the antibacterial activity, obtained from Merck.

### 2.2. Obtaining Chilean Natural Nanozeolite, Reduction Processes

The first stage, a dry size reduction, was carried out in a ball mill (LABTECH HEBRO, Santiago, Chile) at 66 rpm for 15 continuous hours [20,21]. During the second stage, the sample was placed into a SUPER MASSCOLLOIDER (MASUKO) equipment. This milling was also in a dry condition, with 10 cyclic repetitions. Different disc openings (1 to 2 μm) were used at a speed of 1500 rpm [22]. For the last stage, the sample was exposed to microfluidization in the Microfluidizer LM10 equipment (Microfluidics^TM^, Massachusetts, MA, USA). The sample was diluted in deionized water at 5% *w/v*. Subsequently, it was passed eight times by the microfluidizer at a pressure of 1350 bar [23].

The resulting solution was centrifuged in a YINGTAI INSTRUMENT ultracentrifuge at 12,000 rpm for 30 min at 18 °C. Finally, the sample was lyophilized for 24 h in the CHRIST BETA 1-8 LD equipment (ANFF, Queensland, Australia). The obtained nanozeolite powder was stored in a desiccator until further use. This sample was identified as nZ.

### 2.3. Ionic Exchange with Copper Salt to Nanozeolite

Before the ion exchange, the nZ sample was subjected to a chemical treatment that allows an increase in surface area by cleaning pores and channels. This procedure was reported by Valdés et al. (2012) and Poblete Olivares (2013), who used natural zeolite as a catalyst. First, the nZ sample was placed in a container with an HCl solution (2.4 M), using a Z:HCl ratio of 1:100. The solution was stirred at room temperature at 22 °C for 24 h. After that, it was centrifuged in the YINGTAI INSTRUMENT equipment at 12,000 rpm for 30 min at 18 °C. Subsequently, it was washed with 1 L of deionized water, and it was centrifuged again. The resulting sample was dried for 24 h at 125 °C [24,25]. This sample was named nZH.

In order to incorporate the copper ions (Cu^2+^) within the structure of the nZ sample, it was necessary to perform an ion exchange between the nZH and the copper salt. This procedure followed the one proposed by Poblete Olivares (2013) and Tekin and Bac (2016), who used zeolite as a delivery vehicle for copper ions (Cu^2+^). In the ion exchange process, 1 g of modified nZH sample was put in contact in 100 mL of an aqueous solution of Cu(NO_3_)_2_·3H_2_O 0.1 M, adjusting the pH to 6. The mixture was kept under magnetic stirring for 24 hours and then centrifuged at 12,000 rpm for 10 min at 18 °C. Thereafter, the sample was washed with 1 L of deionized water and centrifuged. Finally, it was dried for 24 h at 125 °C [12,25]. The samples were stored in a desiccator until later use. The obtained sample was registered as nZH-Cu.

### 2.4. Characterization Methods for nZ, nZH, and nZH-Cu

The morphology of the nanoparticles was characterized by scanning electron microscopy and elemental surface analysis (SEM-EDX), in a BRUKER QUANTAX EDS XFlash^®^ 6 apparatus (BRUKER, Billerica, MA, USA). In this elemental quantification analysis, the content of Al and Si in the nZ sample was measured. The morphology and dimensions of the nZ sample were evaluated by an Atomic Force Microscope (Naio AFM, Nanosurf AG, Liestal, Switzerland). A histogram of the nZ size distribution was obtained, using the Image J software. The elementary quantification of copper on the surface of the nZH-Cu sample was carried out by SEM-EDX elemental analysis.

The surface area and pore size distribution of the Z, nZH, and nZH-Cu samples were determined by the nitrogen adsorption method at −196 °C, using registered data of *P/Po* (0–0.15). This analysis was carried out in a Micromeritics Gemini 2370 equipment [24].

The evaluation of the nZ sample crystallinity was carried out by X-ray diffraction (XRD). For this, samples of unmodified and modified zeolite powder were taken. The profile of the X-ray pattern was made by a Smartlab model Rigaku diffractometer, with Theta-Theta Bragg-Brentano geometry and a D/teX model Ultra 250 solid-state detector (Rigaku Corporation, Tokyo, Japan). The software, PDXL 2 v.2.7.3.0 (Rigaku Corporation, Tokyo, Japan), and the reference database, ICDD 2018 PDF-4 (International Centre for Diffraction Data, Newtown Square, PA, USA), were used for the matching search phases.

Physical characterization of the nZ sample was done by infrared spectrometry by Fourier transform (FTIR). For this, spectra from nZ powder samples without ion exchange with copper salt (only with acid treatment, nZH) and with ion exchange with copper salt (nZH-Cu) were taken, using a Perkin Elmer Spectrum equipment with the attenuated total reflection (ATR) technique.

### 2.5. Microbiological Assays

The methods of study of the in vitro antibiotic sensitivity used in this research were standardized and approved by the National Committee for Clinical Laboratory Standards (NCCLS) [26,27]. The antimicrobial activity of nZH-Cu sample was evaluated against Gram-negative bacteria (*Escherichia coli*, ATCC 25922) and Gram-positive (*Staphylococcus aureus*, ATCC 25923), standardized at 0.5 Mc Farland, approximately 1.5 × 10^8^ CFU/mL. For the disc diffusion and double serial dilution tests, the nZH powder was used as the control.

#### 2.5.1. Disk Diffusion Assay

In this assay, the methodology reported by Tekin and Bac (2016) was conducted. Samples of 1 and 3 mg/mL of nZH-Cu powder was pressed into pellets and placed on inoculated Müller Hinton agar plates with the microorganisms. Subsequently, sterile discs of 0.6 mm in diameter were moistened with 17 μL of sterile distilled water and placed on the pellets [12]. For the positive controls of antibacterial activity, Ciprofloxacin 5 μg (CIP 5) and Cefoxitin 30 μg (FOX 30) (Oxoid ™) antibiotics were used, and a disk with distilled and sterilized water was used as a negative control. Samples of 1 and 3 mg/mL of nZH pellets were used as the control. All samples were incubated at 37 °C for 24 h. Finally, the antimicrobial activities of the sample of nZH-Cu were evaluated by measuring the zone of inhibition in mm.

#### 2.5.2. Determination of the Minimum Inhibitory Concentration and Minimum Bactericidal Concentration

The minimum inhibitory concentration (MIC) and the minimum bactericidal concentration (MBC) of the nZH-Cu were obtained by the turbidity determination assay (double dilution in broth) [28,29]. According to the procedure, two sets of tubes with triplicate broth were prepared, where 3 mg/mL of nZH-Cu was added to the first tube of each set. Once the sample was homogenized, a double serial dilution was made until the fifth tube of each series was reached. Each one of the sets of tubes with decreasing concentrations of nZH-Cu were inoculated at a concentration of 1 × 10^6^ CFU/mL, from an overnight culture of each of the strains. Samples of 1 and 3 mg/mL nZH were used as the control. All samples were incubated at 37 °C for 24 h. Then, bacterial growth was evaluated by observing the turbidity in the tubes.

#### 2.5.3. Colony Count by the Micro Drop Technique

After the determination of MIC and MBC, an aliquot of 10 μL of the sample was seeded from each tube of the set in plates with Müller Hinton agar [29,30]. They were incubated for 24 h at 37 °C. For the colony count, the following equation was used:

(1) $$\mathrm{Colony}\;\mathrm{count}\;\left(\mathrm{CFU}/\mathrm{mL}\right)=\frac{N{}^{\circ}\;\mathrm{of}{\;\mathrm{colonies}\;*\;\mathrm{dilution}\;\mathrm{factor}}^{*}}{\mathrm{aliquot}\;\mathrm{volume}\;(\mathrm{mL})}.$$

Reciprocal value of the dilution *.

### 2.6. Statistical Analysis

For the analysis of the data and calculation of the results, Microsoft Excel software version 2013 was used. The data are presented as the arithmetic mean with their respective standard deviations. In the case of the disc diffusion assay, a *t*-test was performed for the means of two paired samples (α = 0.05). With this test, it was possible to show if there were significant differences between the two concentrations of nZH-Cu used in the test.

## 3. Results and Discussion

### 3.1. Size Reduction of Natural Zeolite Samples

**Figure 1:**
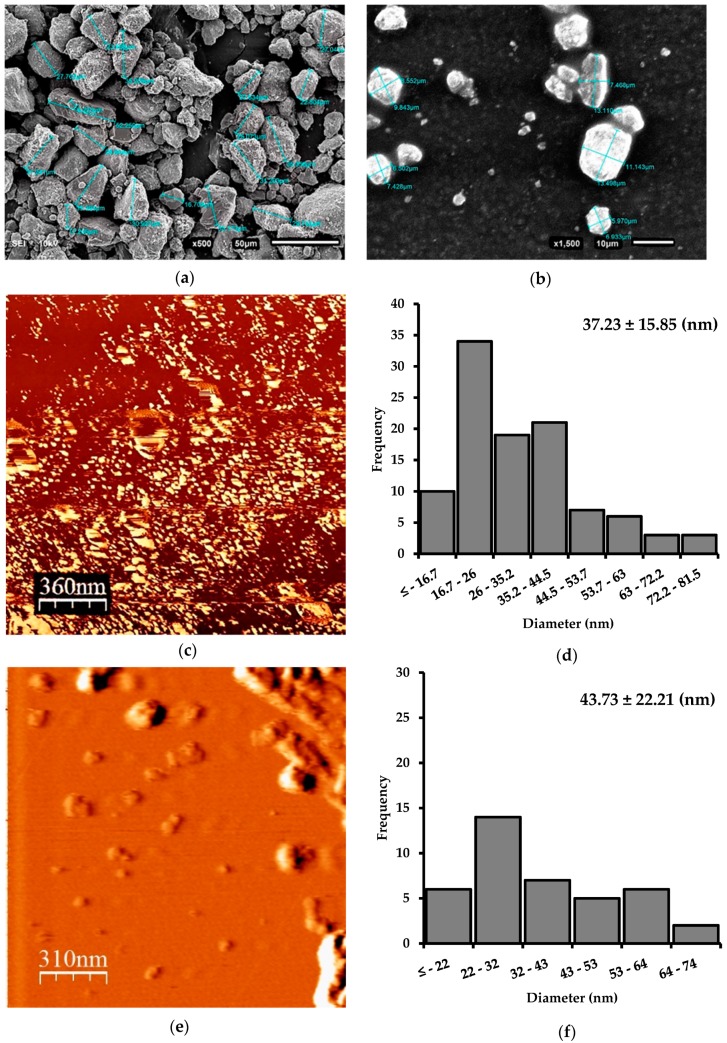
SEM and AFM images of natural zeolite particles after different size reduction processes. (**a**) SEM. Zeolite microparticles after the ball mill process; (**b**) SEM. Zeolite microparticles after the grinding process in a SUPER MASSCOLLOIDER; (**c**) AFM. nZ after the microfluidization process; (**d**) nZ histogram. (**e**) AFM. nZH-Cu; (**f**) nZH-Cu histogram.

The natural zeolite used in this study were provided as small particles of approximately 0.5 to 1.0 cm wide and long. To obtain the natural zeolite nanosamples, three milling stages were necessary. In the first stage, with the use of a ball mill, destructuring of the macroparticle was achieved. As a result, primary rupture products with irregular shapes of different sizes were obtained (Figure 1a). Ozkan et al. (2009) indicate that it is challenging to reach ultrafine sizes by dry milling in a ball mill. This may be due to a ball coating or a bed of fine cohesive particles that develop almost liquid properties so that the particles flow away from the ball–ball collision region and insufficient stress is transmitted to the individual particles to form a fracture [21]. The size reduction process was continued using a SUPER MASSCOLLOIDER (MASUKO). The reduction by grinding with cyclic repeats allowed fractions of zeolite of the micrometric order to be obtained (Figure 1b). The last stage of grinding was carried out in a Microfluidizer LM10. Wet grinding (5% *w/v*) efficiently reduced the zeolite microparticles to nZ. Microfluidization, as a method of reducing the size at high energy, can achieve a uniform reduction in particle size [31]. The sample was subjected to high pressure and was forced to pass through an auxiliary processing module, interacting with a chamber of small channels (100 μm). In this way, high current velocities and cutting forces were produced when the sample collided with each other and with the walls of the cannel [23]. As a result, nanometric order sizes were obtained (Figure 1c).

### 3.2. Morphological Characterization of nZ

According to Figure 1a,b, it is evident that the larger zeolite crystals were typically irregular in shape, whereas after the microfluidization process (Figure 1c), the smaller zeolite nanocrystals tended to be spherical. The microfluidization process gave zeolite particles an average width of 37.2 ± 15.8 nm (Figure 1d). The high shear mechanical process was adequate for the production of nZ. Figure 1e shows that the diameter of nZH-Cu particles tended to increase slightly. The average nZH-Cu was 43.73 ± 22.21 nm (Figure 1f). The increase was caused by the union of the copper salts on the nZH, which generated agglomeration between the particles.

### 3.3. Surface Area and Pore Size Distribution of Z, nZH, and nZH-Cu

The BET model was used in the calculation of the surface area for the Z, nZH, and nZH-Cu samples. After acid treatment, the surface area of the nZH sample increased up to 181.5 m^2^/g (4.5 times) (Table 1). This value is relatively lower compared to those reported by Evangelista et al. (2008) and Valdés et al. (2012) [24,32]. However, they used harsher acid treatments to clean the pores of the natural zeolite. In our research, we used a mild acid treatment (2.4 M HCl). According to the analysis of Table 1, for the nZH sample, the volume of micropores increased from 0.005 to 0.0506 cm^3^/g (10 times) and the pore diameter decreased from 14.6 to 9 nm, after the acid treatment. This phenomenon was caused by the breakage of some larger pores to generate smaller ones. It was observed that acid treatment significantly influenced the surface area of the natural zeolite samples. After ion exchange, the surface area of the nZH-Cu sample decreased to 41.5 m^2^/g, the volume of the micropores decreased to 0.005 cm^3^/g, and the pore diameter increased slightly to 11.15 nm. The explanation for this phenomenon is that the copper salts adhered to the structure of the nZH sample.

This phenomenon is similar to the one previously reported by Baghbanian et al. (2014). Baghbanian et al. synthesized palladium nanoparticles on natural nanozeolite (clinoptilolite). Their results showed that the specific surface of the natural nanozeolite (clinoptilolite) increased from 49.7 to 55.6 m^2^/g, and the pore diameter decreased from 12.19 to 8.05 nm, after acid activation. However, after the coupling of palladium in nanozeolite, the BET surface area decreased and the pore diameter tended to increase [33].

### 3.4. EDX Analysis to nZ, nZH, and nZH-Cu

**Figure 2:**
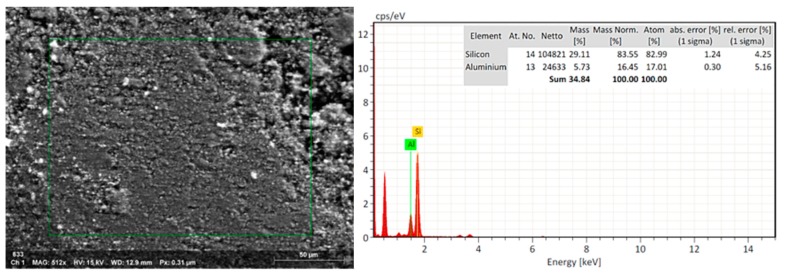
SEM-EDX. EDX analysis of the nZ sample without treatment.

**Figure 3:**
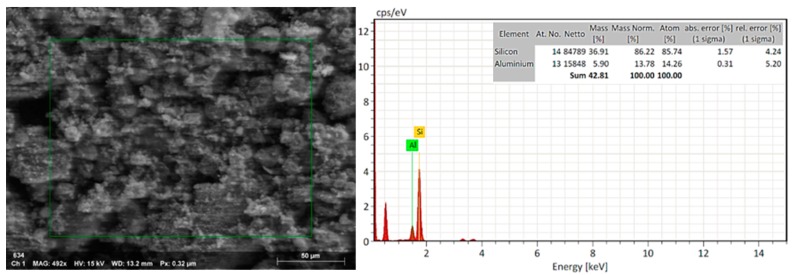
SEM-EDX. EDX analysis to nZ after acid treatment (nZH sample).

The natural zeolite was subjected to an acid treatment to clean the channels in which copper salts could then be added. The EDX analysis of the nZ sample indicated that the Si/Al ratio changed from 5.08 (29.11/5.73) to 6.26 (36.91/5.90) after the acid treatment (Figure 2 and Figure 3). This is a consequence of the acid treatment due to the removal of the aluminum from the structure of the natural zeolite by a decationization and dealuminization process. When comparing this result with the work done by Alejandro (2013), it infers that there is a similar proportion in the change of the Si/Al relation. Alejandro (2013) reported a change in the Si/Al ratio from 5.34 to 7.09 after acid treatment. It was reported that the pH influences the properties of active surface sites. The acid treatment led to an increase of the acid active sites in the metal oxides of the surface of the zeolite [24,34].

**Figure 4:**
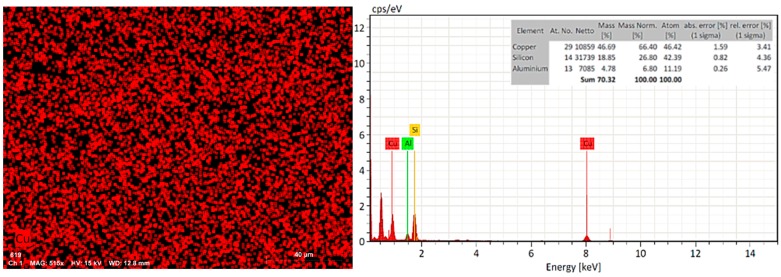
SEM-EDX. EDX analysis to nZ after the ion exchange process (nZH-Cu sample).

Due to the increase of the surface area of the nZ sample, the ion exchange with copper salt led to a coupling on the pores of the zeolite. The result of the EDX elemental analysis indicated that, after the ion exchange treatment with Cu(NO_3_)_2_*3H_2_O 0.1 M for 24 h at room temperature, practically half (46.69%) of the nZH-Cu sample was copper (Figure 4). The sodium molecules exchanged with the copper ions (Cu^2+^) in the zeolite during the wet impregnation. It has been shown that the use of zeolites exchanged with copper ions (Cu^2+^) gives antibacterial activity to natural zeolite. Copper-based nanomaterials have a low cost of source materials, negligible sensitivity to human tissues, and high sensitivity to microorganisms [12,35].

### 3.5. Analysis of Crystal States to nZ, nZH, and nZH-Cu

**Figure 5:**
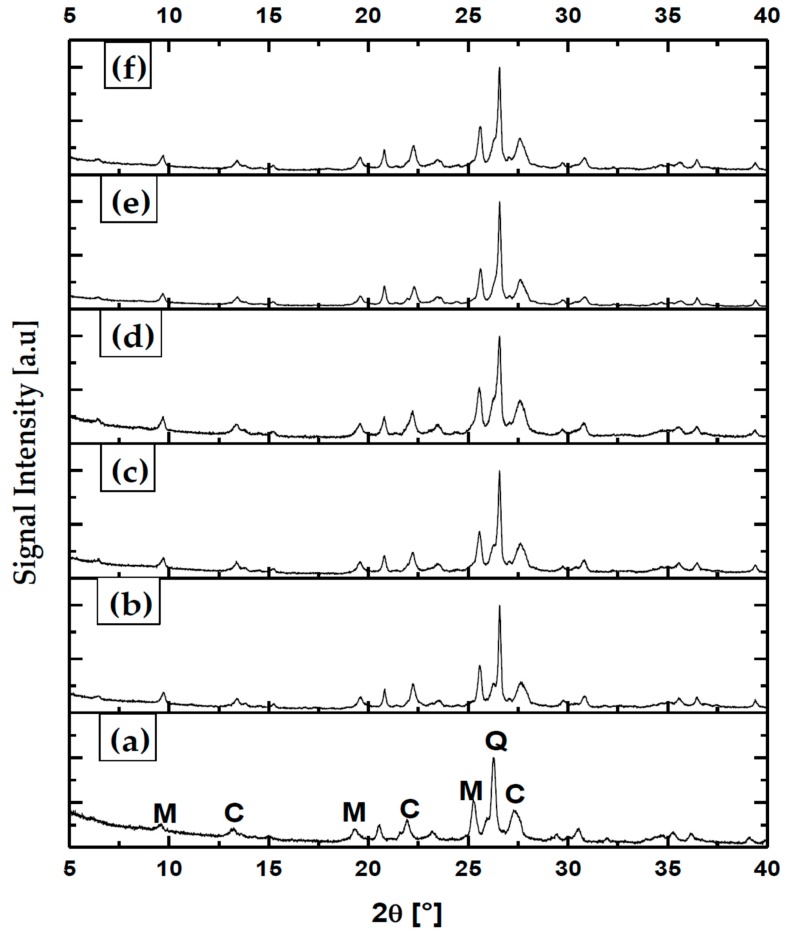
XRD to samples of natural zeolite. (**a**) Z without grinding; (**b**) Z first grinding; (**c**) Z second grinding; (**d**) nZ; (**e**) nZH; (**f**) nZH-Cu.

The natural zeolites that Chile possesses are mostly a mixture of clinoptilolite and mordenite, and contain smaller quantities of quartz. These zeolites are of high purity [4,36]. In fact, the results of XRD to the treated samples, coming from the Minera Formas, confirm this. Figure 5 indicates that the sample of natural zeolite is highly crystalline. Characteristic peaks of clinoptilolite (C), mordenite (M), and quartz (Q) were identified in the zeolite samples. There is evidence of an increase in the width of peaks for M, C, and Q, caused by the grinding processes. For M and C, there is a slight decrease in peak intensity. Therefore, the grinding process causes a decrease in intensity and increases the peak width, from the first grinding process (Figure 5b) and consecutively [37]. For the nZH-Cu sample, the largest peak width is recorded, which indicates nanometric dimensions (Figure 5d).

The comparative analysis between samples of zeolite subjected to different methods of size reduction and chemical treatments indicated that the nZ samples have a high stability in their crystalline structure. This could possibly be attributed to the fact that the treatments were carried out with low concentrations and temperatures. The assignment of the bands of the average infrared zone to the structural vibrations of the natural Chilean zeolite has been widely discussed by Alejandro et al. (2011) and Valdés et al. (2012). When being compared, it can be deduced that they possess similar crystalline structures [24,34]. The thermal and chemical stability depends on several factors, among them the type of structure and the composition of the natural zeolite. The deterioration of the structure of the zeolite is affected by strong acids, mainly with harsh treatments. Unlike the Y, Am and X zeolites, which were destroyed by acid treatments, zeolites, such as mordenite and clinoptilolite, are more resistant, reaching total protonation before being destroyed [38].

### 3.6. Physical Characterization of nZH and nZH-Cu using FTIR

**Figure 6:**
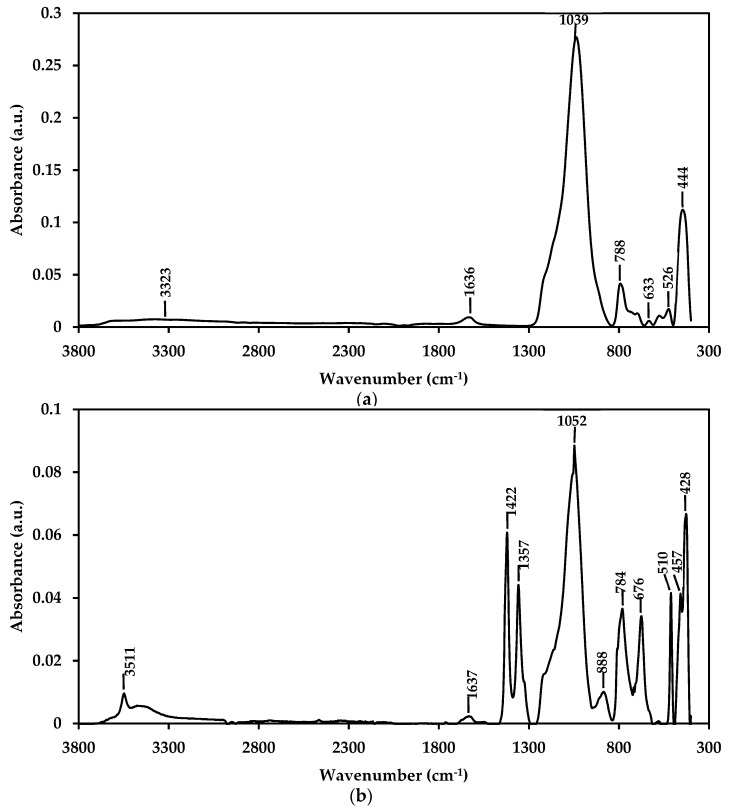
FTIR spectra of samples of natural zeolite. (**a**) nZH; (**b**) nZH-Cu.

Figure 6 shows the spectra obtained from the samples of nZH and nZH-Cu. Figure 6a indicates that the peak for the samples without ion exchange are 3323, 1636, 1039, 788, 633, 526, and 444 cm^−1^. When compared, these peaks present similarities to the absorbance of pure zeolite shown in the work of Delmás et al. (2009) and Shoja et al. (2012) [39,40].

According to Figure 6b, for the nZH-Cu sample, it can be seen that new peaks are added. The band at 3511 is attributable to the stretching of hydroxyl groups (O–H) [35,40,41]. The bands at 1422, 1357, and 888 cm^−1^ explain the ion exchange produced between the sodium ion and the copper ion (Cu^2+^), indicating the addition of copper salts on the zeolite frames. This result is comparable to that reported by Liu et al. (2011). The displacement of the length in the peaks and the appearance of new ones may be due to the interaction of the zeolite and the copper network. According to Liu et al. (2011), these changes are produced by distortion or interaction in the zeolite networks. The interaction is generated by electrostatic forces between the negative matrix and the cations, which accounts for the ion exchange in the sample [41].

### 3.7. Microbiological Assays

#### 3.7.1. Disk Diffusion Assay

**Figure 7:**
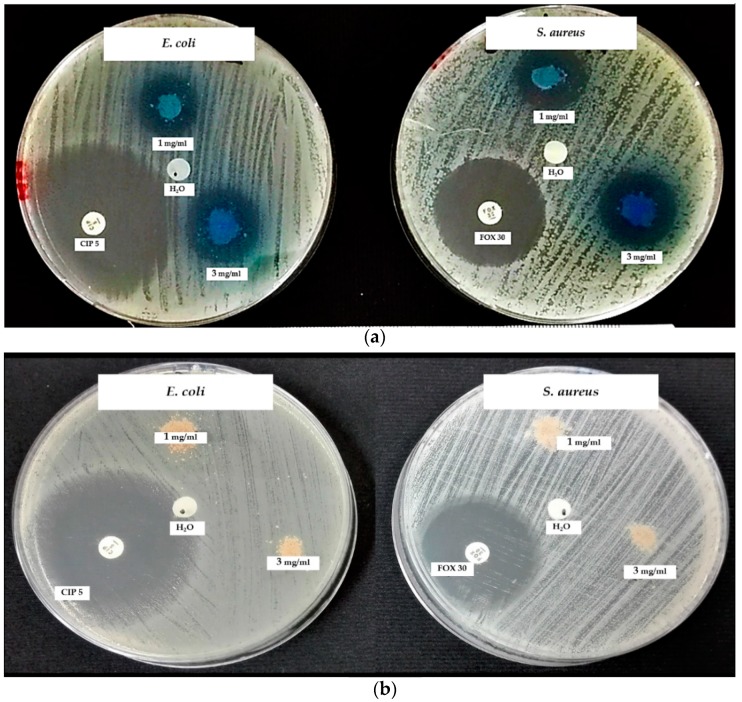
Disc diffusion assay against *E. coli* and *S. aureus*. (**a**) Using 1 and 3 mg/mL of nZH-Cu as an antimicrobial agent. (**b**) Using 1 and 3 mg/mL of nZH, *n* = 3.

Figure 7 shows the results obtained in this work from the disc diffusion assays. Figure 7a shows that the zone of inhibition against *S. aureus* (23–24.7 mm) is relatively larger than that for *E. coli* (20.2–23.2 mm). Figure 7b shows the plates incubated for the disk diffusion assays, with 3 and 1 mg/mL of nZH sample against both strains. As expected, the nZH sample, since it does not carry copper ions, had no antibacterial action. Natural zeolite by themselves are not effective as an antibacterial but function as a support for ions that can be antibacterial [8].

**Figure 8:**
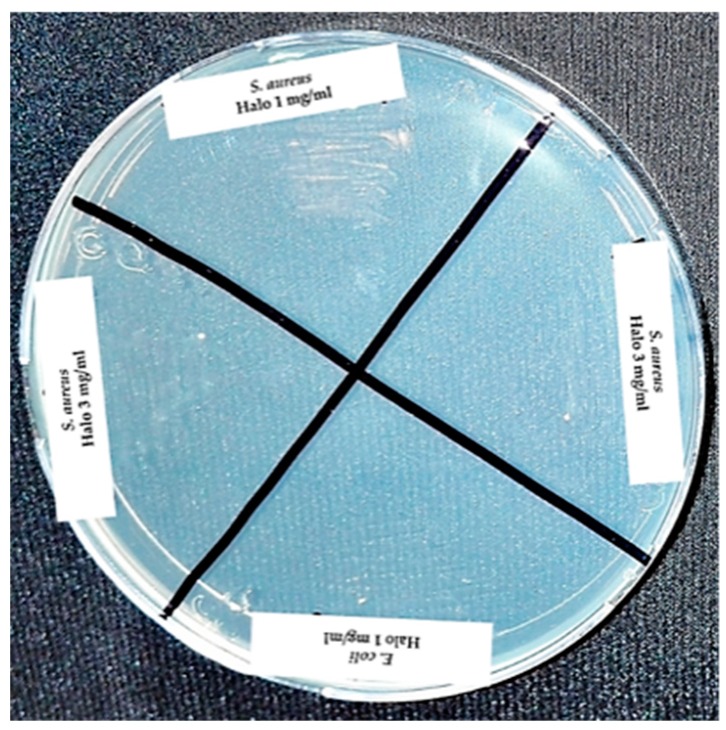
Seeding from the halos of inhibition for the strains of *E. coli* and *S. aureus* against nZH-Cu.

By sowing the inhibition halos produced by the antibacterial, it was confirmed that the concentration of 1 and 3 mg/mL of nZH-Cu sample possessed bactericidal activity toward *E. coli* and *S. aureus* in both concentrations (Figure 8).

Table 2 shows the results obtained regarding the disc diffusion assays and their classification of antimicrobial sensitivity as established by the Clinical and Laboratory Standards Institute. For the *E. coli* strain, the antibacterial with a concentration of 1 mg/mL of the nZH-Cu sample had an intermediate classification, and the antibacterial with a concentration of 3 mg/mL of the nZH-Cu sample was classified as susceptible. In the case of *S. aureus*, both concentrations of the antibacterial proved to be susceptible [26].

In the study conducted by Tekin and Bac (2016), the zeolite X was used as a support for copper salt ions. To dope the zeolite X, they used ion exchange at a concentration of 1 M of CuSO_4_, combined with 80 g of zeolite X per liter of solution. The results of their assays produced an inhibition halo of 17 mm for *E. coli* and 20 mm for *S. aureus* [12].

Alswat et al. (2017) used a co-precipitation method to prepare copper oxide and zeolite nanocomposites. They used a ratio of CuO nanoparticles of 1%, 3%, 5%, 8%, and 10% by weight loaded in the zeolite. Tests of the antibacterial activity indicated that halos of inhibition of 17 mm were obtained for *Bacillus subtilis* and 14.5 mm for *Salmonella choleraesuis* [13].

Milenkovic et al. (2017) studied the antibacterial activity of natural and synthetic zeolite A, doped with ions of Ag, Cu, and Zn, against the *E. coli* strain. The results indicated that the antibacterial activity started from the first hour of contact with the sample. The leaching tests applied in the study indicated that the bactericidal action was due to the metal ions used as bactericidal agents [2].

The result of the *t*-test for the means of the two paired samples shown in Table 3 indicates that against both strains, the statistical value, t (*E. coli*: −3, *S. aureus*: −0.76), is less than the critical value (4.3). Therefore, they do not present significant differences. This statistical result allows the inference that for each strain, the average halo diameter is equal in the two doses of the nZH-Cu sample (1 year 3 mg/mL). Thus, both doses cause the same effect. Therefore, it is possible to use 1 mg/mL of nZH-Cu to obtain a halo of inhibition against *E. coli* and *S. aureus* (Gram-negative and Gram-positive bacteria, respectively). It is important to note that the concentrations of the nZH-Cu sample (1 and 3 mg/mL) used in this study have 46% copper (according to the EDX analysis, Figure 4). Therefore, the bactericidal action of the nZH-Cu sample powder corresponds, approximately, to half its weight.

Gram-positive and Gram-negative bacteria have different susceptibility concerning nanocomposites with antibacterial agents [42]. Gram-positive bacteria possess a thick layer of peptidoglycan; Gram-negatives have a layer of peptidoglycan of a lower proportion and an outer membrane associated with lipopolysaccharides. The lower proportion of peptidoglycan and the negative charge associated with lipopolysaccharides could have a higher affinity to positively charged ions, as in this case. This interaction would allow a higher association and incorporation of ions through the outer membrane of Gram-negative bacteria, with the subsequent cellular damage described in the literature. However, the effects of this type of compound on microorganisms depend on the type microorganism, type of compound, physical or chemical factor, concentrations, and physiological state of the bacteria [17,18].

#### 3.7.2. Determination of the MIC

**Figure 9:**
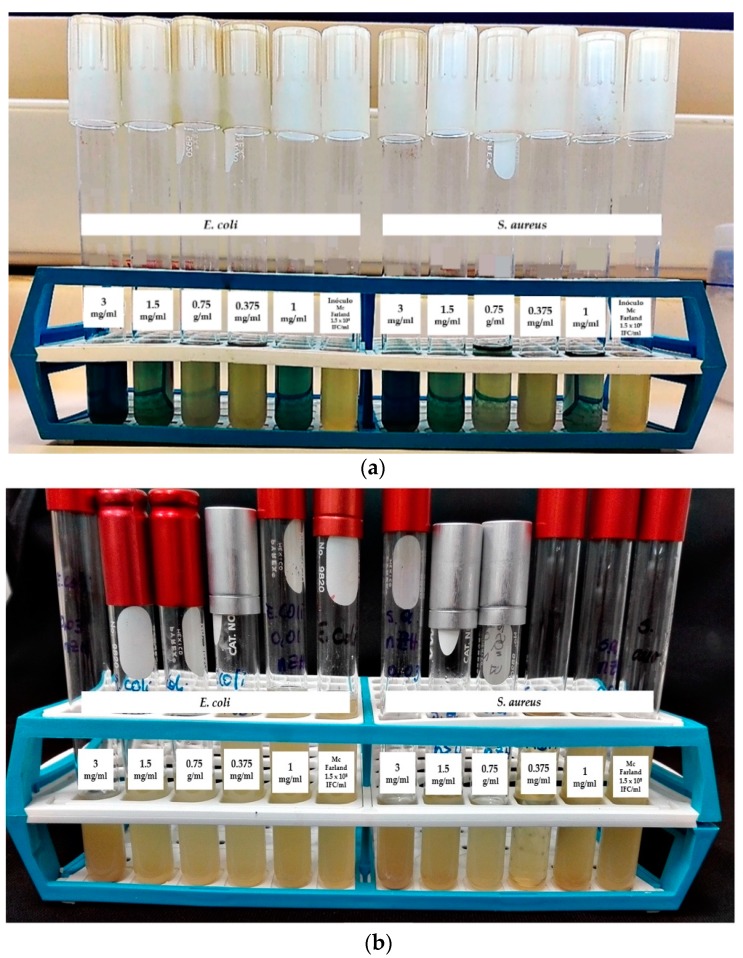
Determination of the MIC by double serial dilution against *E. coli* and *S. aureus*. (**a**) Using 3 and for 1 mg/mL of nZH-Cu. (**b**) Using 3 and 1 mg/mL of nZH.

Figure 9 shows the results obtained in this work from the determination of the MIC by double serial dilution against *E. coli* and *S. aureus*.

Table 4 shows the results of the MIC, MBC, and bacterial count. In the assay for the determination of turbidity by serial double dilution, for the *E. coli* strain, it was observed that with concentrations of 3, 1.5, and 1 mg/mL of the nZH-Cu sample, there was no turbidity. This means that there was no bacterial growth. The MIC achieved with 1 mg/mL of the nZH-Cu sample was verified, due to the fact that 0.75 mg/mL of the nZH-Cu sample showed turbidity (Figure 9a). For the *S. aureus* strain at the concentrations of 3, 1.5, 0.75, and 1 mg/mL of the nZH-Cu sample, no bacterial growth was observed. An MIC was obtained with 0.75 mg/mL of the nZH-Cu sample, while with 0.375 mg/mL of nZH-Cu, no turbidity was observed (Figure 9a). From Figure 9b, turbidity was observed in the trials when using the nZH sample, which indicates bacterial growth.

#### 3.7.3. Determination of MBC and Colony Count Using the Microdrop Technique

**Figure 10:**
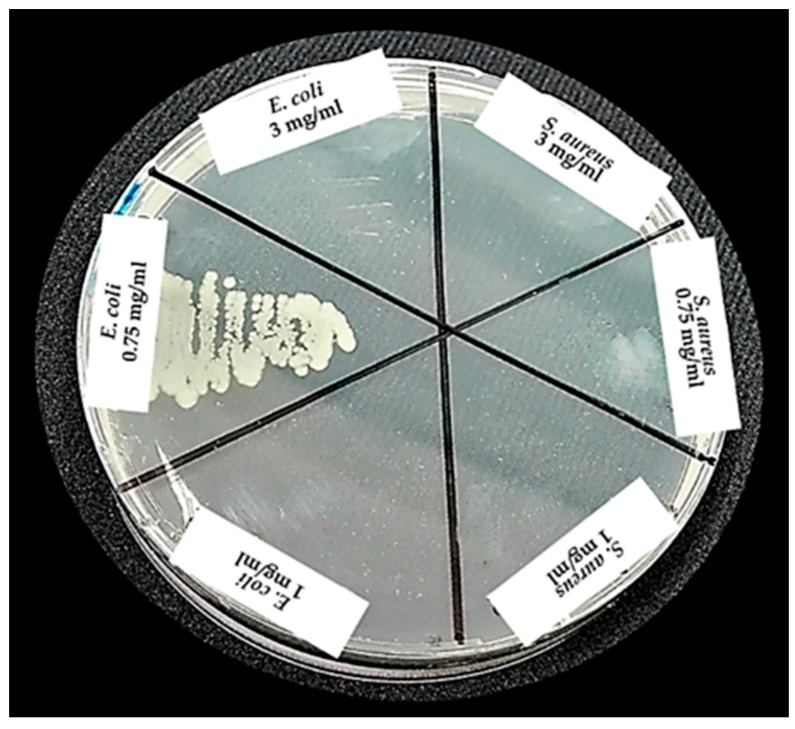
Sowing from the tubes with strains of *E. coli* and *S. aureus* at concentrations of 3, 0.75, and 1 mg/mL of nZH-Cu.

For the determination of the MBC, a sample of the tubes with both strains at concentrations of 3, 0.75, and 1 mg/mL of the nZH-Cu sample was seeded on a plate with Müller Hilton agar (Figure 10).

According to the observations, bacterial growth in the tube with *E. coli* and 0.75 mg/mL of the nZH-Cu sample occurred. Therefore, the 1 mg/mL nZH-Cu sample concentration is bactericidal against the *E. coli* strain. In the case of *S. aureus*, there was no bacterial growth with 0.75 mg/mL of the nZH-Cu sample. Therefore, this concentration is bactericidal against *S. aureus*. According to Febré et al. (2016), copper ions act as a bactericide at concentrations higher than 150 μM, so the bactericidal action of copper ions is a function of the concentration used [16]. In the present study, a concentration of 1 mg/mL was the MBC for *E. coli,* and 0.75 mg/mL was the MBC for *S. aureus*.

**Figure 11:**
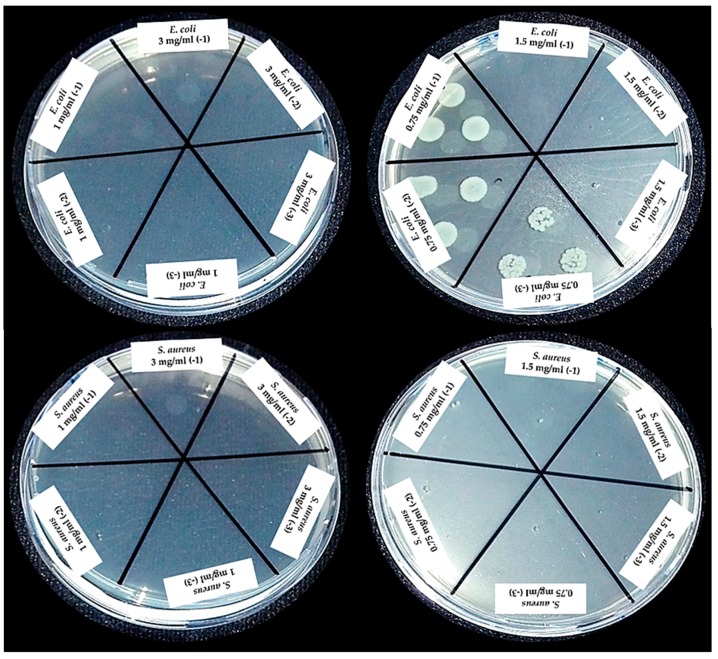
Bacterial count by the microdrop technique and determination of the antibacterial activity of nZH-Cu against reference strains of *E. coli* and *S. aureus*.

Using the colony count test by micro droplets (10 μL of seeding from turbidity sample, with a dilution factor of 1000), it was corroborated that a growth of 6.57 ± 0.05 (Log_10_ UFC/mL) was observed only for *E. coli* at a concentration of 0.75 mg/ml of the nZH-Cu sample as shown in Figure 11. The other concentrations tested in this test were bactericidal.

## 4. Conclusions

The obtainment of Chilean natural nanozeolite doped with copper ions and its antibacterial activity was investigated in this article. After three reduction processes, it was evidenced by AFM that nano-sized particles were obtained. The XRD analysis indicated that the natural Chilean zeolite sample was mostly composed of clinoptilolite and mordenite. The width in the peaks indicated that the particles have nanometric dimensions. It was also shown that the processes of size reduction, acidification, and ion exchange did not significantly affect the crystalline structure of the natural zeolite. It was observed that acid treatment had a significant influence on the surface area of the natural nano zeolite samples. The SEM-EDX analysis of nanozeolites indicated that the acid treatment led to an increase of the Si/Al ratio. SEM-EDX also indicated that the ion exchange process with copper salt produced a high adherence (nZH-Cu, 46% copper). The FT-IR analysis showed new peaks for the nZH-Cu sample, which were associated with the coupling of copper ions in the structure of the nanozeolite. The microbiological tests showed that the zeolite doped with copper salt ions (nZH-Cu) possesses antimicrobial activity with a bactericidal effect against the strains of *Escherichia coli* and *Staphylococcus aureus*, primary pathogens that are present in food and are resistant to multiple drugs. On the other hand, the nZH sample control sample did not show antibacterial activity. The statistical result (*t*-test for means of two paired samples) indicated that it is possible to use 1 mg/mL of the nZH-Cu sample to obtain an inhibition halo against strains, *E. coli* and *S. aureus*. The results obtained from the determination of the MIC by double serial dilution indicated that for the *E. coli* strain, no bacterial growth occurred when concentrations of 3, 1.5, and 1 mg/mL of nZH-Cu sample were used. For the *S. aureus* strain, at the concentrations of 3, 1.5, 0.75, and 1 mg/mL of nZH-Cu sample, no bacterial growth occurred. The colony count test by micro droplets corroborated that only the *E. coli* at a concentration of 0.75 mg/mL of nZH-Cu allowed bacterial growth. The other concentrations tested in this analysis were bactericidal.

The results of this work highlight the potential of natural Chilean zeolite as a copper ion support as a new potential antibacterial nanomaterial. Besides, its nanometric dimensions contribute to increasing the available surface area, allowing its applicability in different matrices.

## Figures and Tables

**Table 1:** Surface area and pore size distribution of Z, nZH, and nZH-Cu.

Sample	S_BET_ (m^2^/g)	BJH Adsorp Average Pore Diameter (nm)	BJH Desorp Average Pore Diameter (nm)	Volume of Micropores (cm^3^/g)
nZ	40.2	14.60	15.73	0.0050
nZH	181.5	9.00	11.62	0.0506
nZH-Cu	41.5	11.15	11.39	0.0050

**Table 2:** nZH-Cu antibacterial activity against reference strains of *E. coli* and *S. aureus*, through disc diffusion assays.

Microbial Strain	nZH-Cu (mg/mL)	Halo (mm)	Classification
***E. coli***	**1**	20.2 ± 0.8 *	Intermediate
	**3**	23.2 ± 1.2 *	Susceptible
***S. aureus***	**1**	23.0 ± 1.1 **	Susceptible
	**3**	24.7 ± 3.6 **	Susceptible

*N* = 3; *, ** mean values without significant differences.

**Table 3:** *t*-test for the means of two paired samples.

Microbial Strain	*E. coli*		*S. aureus*	
nZH-Cu (mg/mL)	1	3	1	3
Mean	20.2	23.2	23	24.7
Variance	0.3	1.3	0.8	14.1
Observations	3	3	3	3
Pearson’s correlation coefficient	–1		0.04	
Hypothetical difference of the means	0		0	
Degrees of freedom	2		2	
Statistic t	−3		−0.76	
P (T <= t) a tail	0.05		0.26	
Critical value of t (one tail)	2.92		2.92	
P (T <= t) two tails	0.10		0.53	
Critical value of t (two tails)	4.30		4.30	

**Table 4:** MIC and MBC of nZH-Cu and colony count through the serial double dilution turbidity determination assay and microdrop technique.

Microbial Strain	Serial Double Dilution of Inoculated Broth and nZH-Cu (mg/mL)	MIC (mg/mL)	MBC (mg/mL)	Count (Log_10_ CFU/mL)
***E. coli***	**3**	No turbidity	Without bacterial growth	Without bacterial growth
	**1.5**	No turbidity	Not rehearsed	Without bacterial growth
	**0.75**	Turbidity	Bacterial growth	6.57 ± 0.05
	**0.375**	Turbidity	Not rehearsed	Not rehearsed
	1 *	No turbidity ^a^	Without bacterial growth ^b^	Without bacterial growth
***S. aureus***	**3**	No turbidity	Without bacterial growth	Without bacterial growth
	**1.5**	No turbidity	Not rehearsed	Without bacterial growth
	**0.75**	No turbidity ^a^	Without bacterial growth ^b^	Without bacterial growth
	**0.375**	Turbidity	Not rehearsed	Not rehearsed
	1 *	No turbidity	Without bacterial growth	Without bacterial growth

* Without dilution; **^a^** MIC; **^b^** MBC.
